# Metabolic engineering of *Phaeodactylum tricornutum* for the enhanced accumulation of omega-3 long chain polyunsaturated fatty acids^☆^

**DOI:** 10.1016/j.ymben.2013.12.003

**Published:** 2014-03

**Authors:** Mary L. Hamilton, Richard P. Haslam, Johnathan A. Napier, Olga Sayanova

**Affiliations:** Department of Biological Chemistry and Crop Protection, Rothamsted Research, Harpenden, AL5 2JQ, UK

**Keywords:** DAG, diacylglycerol, DGAT, acyl-CoA:diacylglyceroltransferase, DHA, docosahexaenoic acid, DPA, docosapentaenoic acid, EPA, eicosapentaenoic acid, LC-PUFA, long chain polyunsaturated fatty acid, MGDG, monogalactosyldiacylglycerol, TAG, triacylglycerol, Diatoms, Genetic engineering, Lipids, Microalgae, Omega-3 long chain polyunsaturated fatty acids

## Abstract

We have engineered the diatom *Phaeodactylum tricornutum* to accumulate the high value omega-3 long chain polyunsaturated fatty acid docosahexaenoic acid (DHA). This was achieved by the generation of transgenic strains in which the Δ5-elongase from the picoalga *Ostreococcus tauri* was expressed to augment the endogenous fatty acid biosynthetic pathway. Expression of the heterologous elongase resulted in an eight-fold increase in docosahexaenoic acid content, representing a marked and valuable change in the fatty acid profile of this microalga. Importantly, DHA was shown to accumulate in triacylglycerols, with several novel triacylglycerol species being detected in the transgenic strains. In a second iteration, co-expression of an acyl-CoA-dependent Δ6-desaturase with the Δ5-elongase further increased DHA levels. Together, this demonstrates for the first time the potential of using iterative metabolic engineering to optimise omega-3 content in algae.

## Highlights

• First example of using metabolic engineering in microalgae to modify the accumulation of high value omega-3 long chain polyunsaturated fatty acids.
• First example of multigene expression in a diatom.
• Detailed lipidomic analysis of diatom *Phaeodactylum tricornutum*.

## 1 Introduction

It is now well established that omega-3 long chain polyunsaturated fatty acids (LC-PUFAs), especially eicosapentaenoic acid (EPA; 20:5∆^5,8,11,14,17^, n-3) and docosahexaenoic acid (DHA; 22:6∆^4,7,10,13,16,19^, n-3) are essential constituents of human nutrition, with key roles in the growth and development of infants and maintaining cardiovascular health in adults (Calder, 2003; Das, 2002; Voigt et al., 2000). Although marine fish are the current main dietary source of EPA and DHA, the depletion of wild fish stocks and pollution of the marine environment (Garcia and Rosenberg, 2010) indicate an urgent need for alternative, sustainable sources of omega-3 LC-PUFAs. Marine microorganisms such as microalgae and diatoms are the primary producers of LC-PUFAs in the aquatic food chain. The cultivation of EPA- and DHA-rich microalgae have been demonstrated as promising alternative sources to fish oils (Harwood and Guschina, 2009; Molina-Grima et al., 1996). Commercial production of high value algal products like omega-3 LC-PUFAs is well-established, with the production (primarily for infant formula and nutraceuticals) of DHA through auxotrophic fermentation representing a multi-billion dollar industry in which supply can barely keep pace with demand. However, this process is expensive to maintain and technologically challenging to expand. Similarly, the feasibility of expressing algal genes in higher plants to direct the non-aquatic production of omega-3 LC-PUFAs has been explored with some success but only limited practical impact (Ruiz-Lopez et al., 2013; Sayanova et al., 2011; Venegas-Caleron et al., 2010). As an alternative approach, there is increasing interest in using metabolic engineering to enhance and redirect the accumulation of omega-3 LC-PUFAs in microalgae, specifically with the goal of elevating the endogenous synthesis of omega-3 LC-PUFAs and their incorporation into neutral lipids such as triacylglycerol (TAG). Currently, no single algal strain accumulates high concentrations of both EPA and DHA in TAG, indicating a clear target for improvement.

**Fig. 1:**
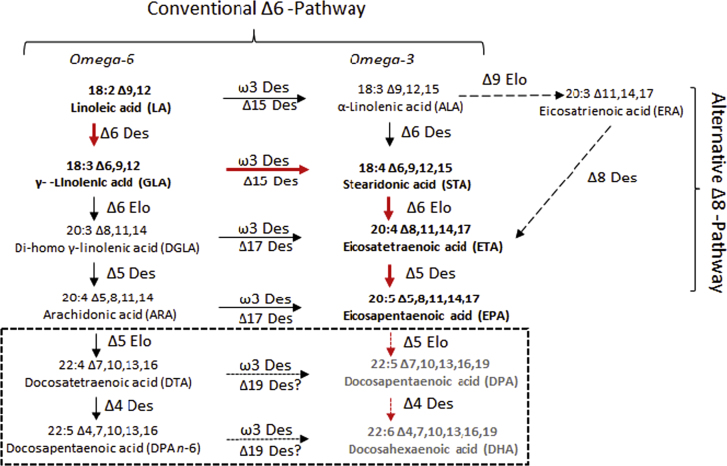
Pathways for the biosynthesis of LC-PUFAs in *P. tricornutum*. The predominant route for the biosynthesis of EPA is shown with red arrows and in bold text, along with other minor routes described by Arao and Yamada (1994). The predicted pathways for the biosynthesis of DHA are within the broken lined box and the expected most active route is shown with red broken arrows and with text in grey. (For interpretation of the references to color in this figure legend, the reader is referred to the web version of this article.)

*Phaeodactylum tricornutum* is a unicellular diatom which accumulates EPA (up to ~35%), but only trace levels of DHA. Considered a good source for the industrial production of EPA (Yongmanitchai and Ward, 1991), it also grows quickly, is easy to genetically transform (Zaslavskaia et al., 2000) and has a fully sequenced genome (Bowler et al., 2008), making it an appealing system for further study. In *P. tricornutum*, the majority of EPA accumulates in polar lipids, especially in galactolipids such as monogalactosyldiacylglycerol (MGDG), as opposed to the more desirable (in terms of extraction and downstream processing) neutral lipids such as TAG (Arao et al., 1987; Tonon et al., 2002; Yongmanitchai and Ward, 1993a, 1993b). Using in vivo labelling, it was shown that EPA can be synthesised in *P. tricornutum* by a number of different routes (Arao and Yamada, 1994) with the predominant route proceeding via Δ6-desaturation of linoleic acid (summarised in Fig. 1). The synthesis of EPA in *P. tricornutum*, involving both Δ6- and Δ5-desaturation (Domergue et al., 2002) and Δ6-elongation, takes place in the endoplasmic reticulum and newly synthesised EPA is then imported into the plastids for incorporation into galactolipids. Evidence from the heterologous expression of *P. tricornutum* desaturases in yeast indicates that these are phospholipid-dependent activities, meaning that the so-called substrate-dichotomy bottleneck exists in this diatom (Domergue et al., 2003; Napier 2007). The *P. tricornutum* lipidome contains only minor amounts of fatty acid intermediates on the EPA biosynthetic pathway (Fig. 1) indicative of a highly efficient mechanism for the accumulation of EPA as a single end-product (Arao and Yamada, 1994). In microalgae, diatoms and protists, DHA is synthesised via the elongation of EPA to docosapentaenoic acid (DPA; 22:5∆^7,10,13,16,19^, n-3) through the activity of a Δ5-elongase, with DPA then converted to DHA by a ∆4-desaturase (Qiu et al., 2001; Meyer et al., 2003). The molecular identity of these two genes in *P. tricornutum* has not yet been confirmed, with DHA levels in this diatom very low (Yongmanitchai and Ward, 1993a, 1993b).

In this study we have used biolistic transformation to overexpress two different genes encoding enzyme activities of the LC-PUFA biosynthetic pathway, namely the Δ6-desaturase and Δ5-elongase, in the model diatom *P. tricornutum.* We demonstrate that the heterologous expression of a Δ5-elongase from *O. tauri* results in significantly increased accumulation of DHA in *P. tricornutum* while co-expression with an acyl-CoA-dependent Δ6-desaturase further increased DHA levels. As an example of metabolic engineering, this study provides definitive and novel evidence for the efficacy of expressing heterologous genes as a means of enhancing the LC-PUFA biosynthetic pathway in transgenic microalgae.

## 2 Materials and methods

### 2.1 Strains and growth conditions

*P. tricornutum* UTEX 646 was grown in ESAW medium (Harrison et al., 1980) at 20 °C with moderate shaking under white fluorescent lights in constant illumination (60 µmol photons m^−2^ s^−1^). Analysis of the wild-type and transgenic algae have been performed during exponential and stationary growth phases. Growth stage was determined by cell counting with reference to growth curves measured for Pt_WT and transgenic cells at 16 °C and 20 °C.

### 2.2 Plasmid design and cloning

The coding sequences for *O. tauri* Δ6–desaturase OtD6 (Domergue et al., 2005) and *O. tauri* Δ5-elongase OtElo5 (Meyer et al., 2004) were inserted as *Kpn-Xba* and *EcoRV-SacI* fragments, respectively, into pPha-T1 vector (Kroth, 2007; Zaslavskaia et al., 2000). The coding region of OtD6 was used as a template to chemically synthesise (Genscript Corporation, NJ) codon-optimised nucleotide sequence OtD6PT for expression in *P. tricornutum*. This codon-optimised Δ6 – desaturase sequence was cloned into pPha-T1 vector, using *EcoRV-SacI* sites.

### 2.3 Design of double-gene vector pPhOS2 and transformation cassettes

The EcoRI – HindIII fragment of of pPha – T1 vector containing MCS was replaced by the synthetic sequence comprising of *fcpA* terminator and *fcpA* promoter flanked by 3 multiple cloning sites (MCSs) with unique restriction sites (Supplementary Fig. 1). The coding sequences for *O. tauri* Δ5-elongase OtElo5 was inserted as *KpnI-SacI* fragment into position 1 of pPhOS vector generating pPhOS2.1.1 construct. The codon optimised coding sequences for *O. tauri* Δ6–desaturase OtD6Pt was inserted as *BamHI-XbaI* fragment into position 2 of pPhOS2.1.1 generating pPhOS2.2.1 construct.

### 2.4 Biolistic transformation of *P. tricornutum*

Biolistic transformation of *P. tricornutum* was performed according to methods previously described (Kroth, 2007). Bombarded cells were transferred onto ESAW agar plates containing 75 µg/ml zeocin. The zeocin plates were placed in 24 h light under fluorescent lights (60 µmol m^−2^ s^−1^) and incubated at 20 °C for 3 weeks. Selected zeocin-resistant colonies were transferred to fresh zeocin plates and 2 ml ESAW plus zeocin cultures before being transferred to liquid medium minus antibiotic for lipid analysis.

### 2.5 Fatty acid analysis of *P. tricornutum*

Algal cells were harvested by centrifugation at 3500*g* for 5 min. Lipids were extracted and methylated as described (Garces and Mancha, 1993) with minor modifications. A 15 ml aliquot of algal culture was harvested; following methylation the heptane fraction was concentrated and re-suspended in 40 µl solvent prior to injection of 1 µl on to the GC column. Methyl ester derivatives of total fatty acids extracted were analysed by GC (Agilent 7890A) using an Agilent DB-225 column (30 m×0.32 mm×0.3 µm). Inlet and detector temperature was set to 250 °C and 1 µl of each sample was analysed using splitless injection and a constant flow rate of 2 ml/min. The oven temperature cycle was set as follows: a start temperature of 50 °C was held for 1 min to allow vaporised samples and the solvent (hexane) to condensate at the front of the column. Oven temperature was then increased rapidly to 190 °C at a rate of 40 °C/min followed by a slower increase to 220 °C at a rate of 1.5 °C/min. The final temperature of 220 °C was held for 1 min giving a total run time of 25 min 50 s per sample. FAMEs were detected using a Flame Ionisation Detector (FID). Chromatograms were analysed using the offline session of the Agilent ChemStation software. The retention time and identity of each FAME peak was calibrated using the FAME Mix Rapeseed oil standard (Supelco) supplemented with 2% w/w methyl 11,14-eicosadienoate(C20:2 n-6). 1 mM Methyl heptadecanoate (C17:0) was added to samples as an internal standard.

### 2.6 Acyl-CoA profiling

Algal cells were harvested by centrifugation (as above), frozen in liquid nitrogen and extracted after Larson and Graham (2001) for reverse-phase LC with electrospray ionisation tandem mass spectrometry (multi reaction monitoring) in positive ion mode. LC-MS/MS+MRM analysis followed the methods described by Haynes et al. (2008). (Agilent 1200 LC system; Gemini C18 column, 2 mm inner diameter, 150 mm with 5 mm particles). For the purpose of identification and calibration, standard acyl-CoA esters with acyl chain lengths from C14 to C20 were purchased from Sigma as free acids or lithium salts.

### 2.7 TAG extraction and MS profiling

TAGs were extracted by a modified Bligh and Dyer (1959) method; cells harvested from 50 mL of culture were heated for 10 min at 95 °C in 1 mL of isopropanol and homogenised using a Potter homogeniser. The homogenate was centrifuged, supernatant collected, and the pellet re-extracted with chloroform:methanol (1:2). Collected fractions were dried down with a stream of nitrogen and re-suspended in chloroform. The molecular species of TAGs were analysed by electrospray ionisation triple quadrupole mass spectrometry (API 4000 QTRAP; Applied Biosystems). Triacylglycerols (TAGs) were measured after Krank et al., (2007) and were defined by the presence of one acyl fragment and the mass/charge of the ion formed from the intact lipid (neutral loss profiling). This allows identification of one TAG acyl species and the total acyl carbons and total number of acyl double bonds in the other two chains. The procedure does not allow identification of the other two fatty acids individually nor the positions (*sn*-1, *sn*-2, or *sn*-3) that individual acyl chains occupy on the glycerol. TAGs were quantified after background subtraction, smoothing, integration, isotope deconvolution and comparison of sample peaks with those of the internal standard (using LipidView™, Applied Biosystems). The profiling samples were prepared by combing 50 μL of the total lipid extract with 950 μL of isopropanol/methanol/50 mM ammonium acetate/dichloromethane (4:3:2:1). Samples were infused at 20 μL min^−1^ with an autosampler (LC mini PAL, CTC Analytics, Switzerland). The scan speed was 100 μ s^−1^. The collision energy, with nitrogen in the collision cell, was +25 V; declustering potential was+100 V; entrance potential was 14 V; and exit potential was +14 V. Sixty continuum scans were averaged in the multiple channel analyser mode. For product ion analysis, the first quadrupole mass spectrometer (Q1) was set to select the TAG mass and Q3 for the detection of fragments fragmented by collision induced dissociation. The mass spectral responses of various TAG species are variable, owing to differential ionisation of individual molecular TAG species. For all analyses gas pressure was set on ‘low’, and the mass analysers were adjusted to a resolution of 0.7 μ full width height. The source temperature was 100 °C; the interface heater was on, +5.5 kV was applied to the electrospray capillary; the curtain gas was set at 20 (arbitrary units; and the two ion source gases were set at 45 (arbitrary units). In the data shown herein, no response corrections were applied to the data. The data were normalised to the internal standards tri15:0 and tri19:0 (Nu-Check Prep, USA).

## 3 Results and discussion

### 3.1 Generation of transgenic *P. tricornutum* over-expressing an acyl-CoA Δ6-desaturase from *O. tauri*

The first committed step in the biosynthesis of omega-3 LC-PUFAs such as EPA and DHA is the Δ6-desaturase (Fig. 1), and this reaction is generally considered to be rate-limiting for this pathway (Sayanova et al., 1997). Moreover, previous studies in transgenic plants and yeast have indicated the importance of acyl-CoA-dependent Δ6-desaturase activities in efficient EPA synthesis, bypassing the metabolic bottleneck of substrate dichotomy between desaturases and elongases (Ruiz-Lopez et al., 2013; Sayanova et al., 2011). We, therefore, attempted to augment the activity of the endogenous *P. tricornutum* phospholipid-dependent Δ6-desaturase via the transgene-derived activity of an acyl-CoA-dependent Δ6-desaturase from the picoalga *Ostreococcus tauri* (Domergue et al., 2005; Petrie et al., 2010). The native coding (OtD6N) and codon-optimised for expression in *P. tricornutum* (OtD6Pt) sequences for the *O. tauri* Δ6-desaturase were cloned into the pPha-T1 vector (Zaslavskaia et al., 2000) and the resulting constructs pPt_OtD6N and pPt_OtD6Pt were used to transform *P. tricornutum* via biolistics. Multiple (>10) independent zeocin-resistant colonies were obtained by transformation with these two expression cassettes and confirmed by further screening. Selected colonies were transferred into liquid medium and grown under constant illumination (60 µmol photons m^−2^ s^−1^) at different temperatures (20 °C and 16 °C) to study the effect of temperature on the synthesis of LC-PUFAs, since previous studies indicated that reduced temperatures (<20 °C) resulted in elevated accumulation of EPA (Jiang and Gao, 2004).

Fatty acid (FA) profiling of wild type *P. tricornutum* (Pt_WT) and zeocin-resistant strains expressing the *O. tauri* desaturases showed that palmitoleic acid (16:1 n-7), EPA (20:5 n-3), palmitic acid (16:0) and oleic acid (18:1 n-9) were the major FAs detected in all algal strains grown to stationary phase (for maximal oil accumulation) at different temperatures. The cell suspensions were collected on the ninth day at the early stationary phase to avoid the effects of advanced age on the cultures (Supplementary Table 1). Fatty acid analysis revealed that in both WT and transgenic cells grown at 16 °C, EPA and DHA levels were higher than for cells grown at 20 °C, in agreement with previous studies (Jiang and Gao, 2004). The levels of EPA and DHA in Pt_OtD6N cells grown at 20 °C in stationary phase were slightly higher than those of Pt_WT (20.0% of EPA and 1.8% of DHA in Pt_OtD6N compared to 18.5% of EPA and 1.3% of DHA in Pt_WT respectively; see Supplementary Table 1). The levels of EPA in Pt_OtD6Pt cells grown at 20 °C in stationary stage were higher than those of PT_OtD6N (20.8% of EPA in Pt_OtD6Pt compared to 20.0% of EPA in Pt_OtD6N). Similar results were observed in WT and transgenic cells grown at 16 °C (22.9% of EPA in Pt_OtD6Pt compared to 22.0% and 20.3% in Pt_OtD6N and Pt_WT respectively; see Supplementary Table 1). Fatty acid profiles from Pt_WT, Pt_OtD6N and Pt_OtD6Pt transgenic *P. tricornutum* showed no differences in accumulation of C18 Δ6-unsaturated fatty acids which were barely present (<2%) (Supplementary Table 1). On the basis of these collective data, we conclude that transgenic over-expression of an acyl-CoA-dependent Δ6-desaturase does not result in statistically significant increased levels of EPA and/or DHA in *P. tricornutum* – the likely explanation for this is discussed below. This is unlike the situation observed in higher plants (in which the Δ6-desaturation step is a rate-limiting bottleneck) (Napier, 2007; Sayanova et al., 2011) and highlights the metabolic differences between the two distinct taxonomic groups (Fabris et al., 2012).

### 3.2 Generation of transgenic algae over-expressing Δ5-elongase from *O. tauri*

**Fig. 2:**
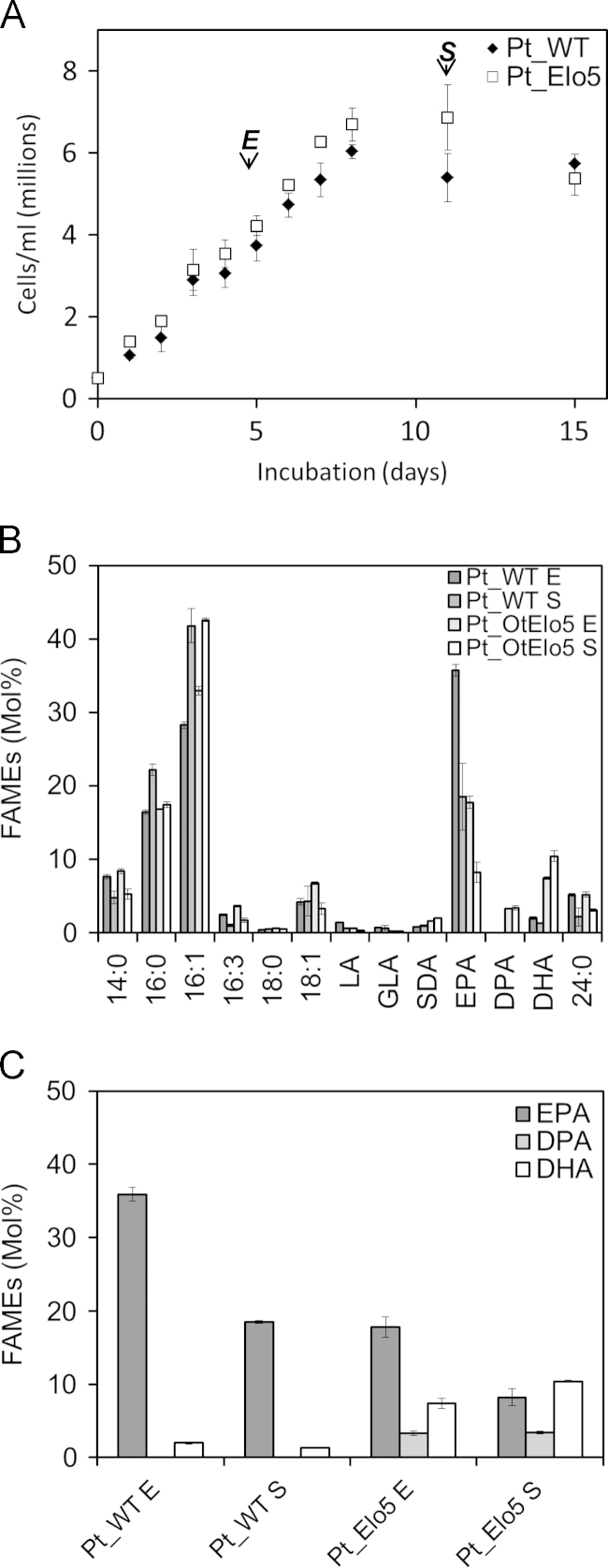
The effect of heterologous Δ5-elongase expression on *P. tricornutum*. (A) Cellular growth of Pt_WT and transgenic *P. tricornutum* expressing the *Ostreococcus tauri* Δ5 elongase cells were harvested for lipid analysis where indicated. (B) Fatty acid composition of Pt_WT and transgenic Pt_OtElo5 cells during exponential (E) and stationary (S) phases. (**C**) EPA and 22 acyl carbon product content in Pt_WT and transgenic Pt_OtElo5 cells. Values are the average of three experiments (± standard error). Growth stage was determined by cell counting with reference to growth curves measured for Pt_WT and transgenic cells at 16 °C and 20 °C.

To determine the biosynthetic fluxes which contribute to the biosynthesis of omega-3 LC-PUFAs, we measured the acyl-CoA pool of wild type *P. tricornutum* at exponential and stationary phases (Supplementary Fig. 2). These data indicated that whilst the *O. tauri* Δ6-desaturase substrates α-linolenic acid (18:3Δ^9,12,15^; n-3) and linoleic acid (LA; 18:2Δ^9,12^) were present at only basal levels in the acyl-CoA pool, EPA-CoA was highly abundant (Supplementary Fig. 2). Therefore, a second approach was taken to engineer the higher accumulation of DHA in *P. tricornutum* via the expression of a C20 Δ5-elongating activity (OtElo5) from *O. tauri* (Meyer et al., 2004). This activity directs the C2 elongation of EPA-CoA to docosapentaenoic acid-CoA (DPA-CoA), the direct precursor of DHA (Fig. 1). Ten zeocin-resistant colonies obtained by transformation of *P. tricornutum* with an expression plasmid containing the native OtELo5 gene were used to inoculate cultures for further screening by GC–MS analysis of fatty acids. Cultures were grown at 20 °C under constant illumination (60 µmol photons m^−2^ s^−1^). The mean levels of DHA in analysed clones ranged from 4.1% to 11% of total fatty acids, against a basal level of <2% in the parental WT strain. Three independent transgenic strains which contained the highest levels of DHA were selected for further analysis. Since differences in analysis of fatty acid composition related to culture age have been observed in microalgae, we generated total fatty acid methyl esters (FAMEs) for analysis by GC–MS/GC-FID during different growth phases. FAMEs were analysed at day five (exponential growth, E) and day 11 (stationary phase, S) (Fig. 2A). The analysis of FAMEs from transgenic *P. tricornutum* strains expressing the OtElo5 C20 elongating activity revealed the presence of DPA in the range of 3.3–3.4%; this fatty acid was not detected in Pt_WT cells (Fig. 2B, Supplementary Table 2). In transgenic strains over-expressing the OtElo5 gene, levels of EPA were decreased to an average of 17.7% (compared to 35.9% in Pt_WT) in the exponential phase of growth and to 8.2% (compared to 18.5% in Pt_WT) during the stationary phase of growth. A concomitant and substantial increase in DHA was observed in Pt_OtElo5 transgenic strains, averaging 7.4% in exponential phase and 10.4% in stationary phase (compared to 2.0% and 1.3% respectively in Pt_WT). DHA accumulation increased upon transition to stationary phase. This represented up to an eight-fold increase in mol% DHA for *P. tricornutum* cells in the stationary (oil-accumulating) phase (Fig. 2C, Supplementary Table 2), mirroring a 7-fold increase in the conversion of EPA to DHA. Apart from the conversion of EPA to DHA and DPA, no other changes to the fatty acid profile were observed (Supplementary Table 2).

### 3.3 Determination of acyl-CoA pool composition in WT and transgenic *P. tricornutum*

**Fig. 3:**
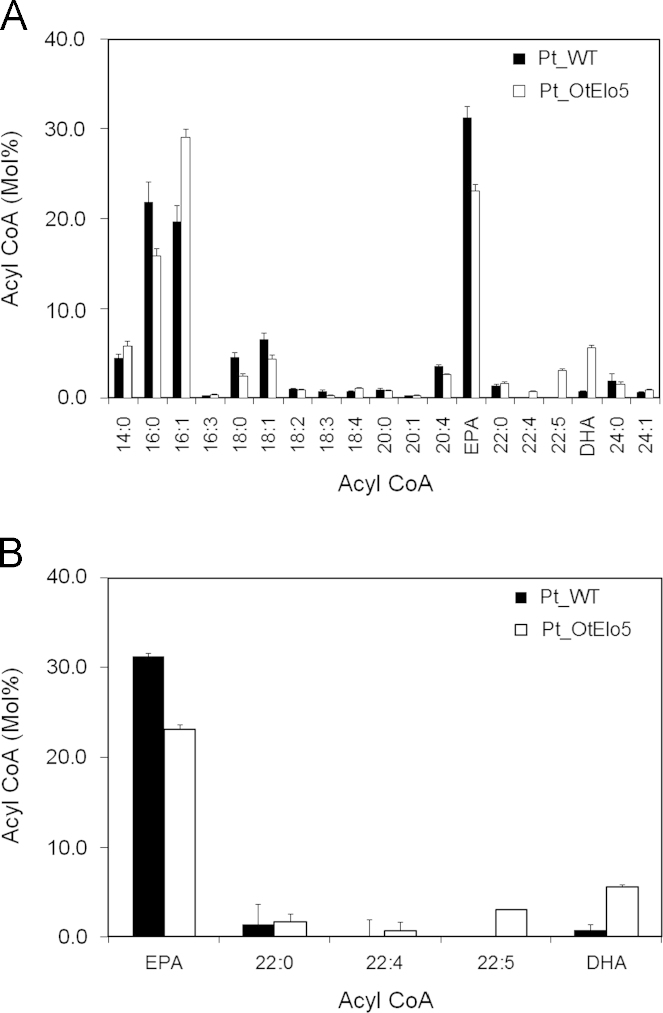
The effect of heterologous Δ5-elongase expression on the acyl-CoA pool of *P. tricornutum*. (A) Acyl-CoA profiles of Pt_WT and Pt_ OtElo5 *P. tricornutum* cells cultures during stationary phase of growth. (B) EPA and selected 22 acyl carbon products from the total acyl-CoA profile. Values are the average of three experiments (± standard error).

To better understand the potential bottlenecks in omega-3 LC-PUFA biosynthesis in diatoms, we compared the acyl-CoA pool composition of the wild-type (Pt_WT) and transgenic (Pt_OtElo5) *P. tricornutum* (Fig. 3A). The acyl-CoA profile of Pt_WT in the stationary phase of growth revealed that palmitic, palmitoleic and EPA (as noted above) were the most abundant fatty acids present in this dynamic metabolic pool, reflecting their predominance in the total fatty acids of *P. tricornutum* (Arao and Yamada, 1994). Interestingly, the relationship is not stoichiometric, since EPA-CoA is the most abundant fatty acid (31.2%) in the acyl-CoA pool for S phase Pt_WT, yet the overall total levels of EPA are significantly lower (18.5%; Supplementary Tables 1, 3). There is a good correlation between the levels of 16:0-CoA and 16:0 in total native fatty acids in the acyl-CoA pool (Fig. 3A) and the total fatty acids observed in Fig. 2B. Only traces (<1.0%) of 22:5 n-3 (DPA-) and DHA-CoAs were detected in the CoA pool of Pt_WT. As can be seen in Fig. 3B, similar analysis of transgenic Pt_OtElo5 *P. tricornutum* cells expressing the OtElo5 activity revealed an altered acyl-CoA profile, with the significant accumulation of 22:5 n-3 and DHA-CoAs accompanied by a decrease in EPA-CoA levels. Unexpectedly, the levels of 16:1-CoAs in the Pt_OtElo5 strains were noticeably higher compared to Pt_WT (Fig. 3A; Supplementary Tables 3) though the reason for this is not obvious (see Fig. 1). Of the C22 LC-PUFA-CoAs, the predominant form is DHA, despite the OtElo5 transgene activity only mediating the conversion of EPA-CoA to the DHA precursor, DPA(-CoA). Thus, the generation of DHA from DPA (by the native *P. tricornutum* Δ4-desaturase) appears to proceed with a good level of efficiency, even though endogenous DHA synthesis is very low (cf. Supplementary Table 1).

### 3.4 Profiling of TAG molecular species present in WT and transgenic *P. tricornutum*

**Fig. 4:**
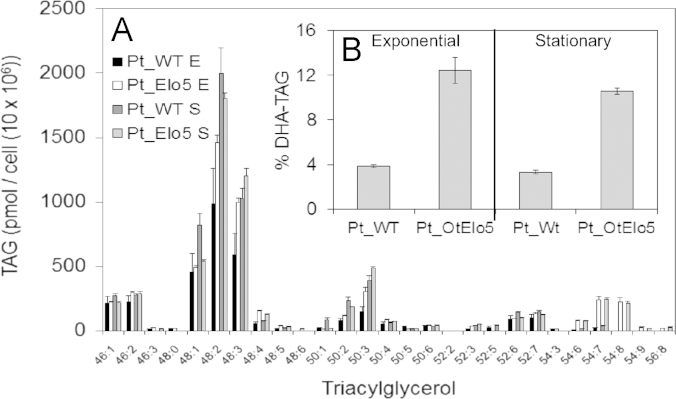
Analysis of triacylglycerol from *P. tricornutum* expressing an *O. tauri* Δ5-elongase. (A) The distribution of TAG species from Pt_WT and transgenic Pt_OtElo5 at different stages of cellular growth. (B) The distribution of DHA in TAG molecular species from Pt_WT and Pt_OtElo5 at different stages of cellular growth. Values are the average of three experiments (± standard error).

The primary objective of our studies was not only to engineer the accumulation of meaningful levels of DHA in *P. tricornutum*, but also to ensure that this high value fatty acid was accumulated in neutral lipids such as TAG (Radakovits et al., 2011). We, therefore, identified and compared the different molecular species of TAGs present in Pt_WT and Pt_OtElo5 strains and also investigated changes in TAG synthesis in response to transition from exponential to stationary phase. Cultures were grown at 20 °C under constant illumination (60 µmol photons m^−2^ s^−1^) and analysed using ESI-MS/MS. The mass spectrum obtained from direct infusion ESI-MS (Supplementary Fig 3) of algal lipid extracts shows that a majority of the molecular ions are observed between 750 and 950 mass/charge (m/z). Further ESI-MS/MS analysis detected 26 individual TAG species in Pt-WT (Fig. 4A; Supplementary Fig. 4). The TAGs of Pt_WT were predominantly composed of compounds of acyl compositions 46:1, 46:2, 48:1, 48:2, and 48:3 and 50:3, i.e. with palmitic (16:0), palmitoleic (16:1), and myristic (14:0) acid substituents on the glycerol backbone. The predominant TAG molecular species 48:1 (16:0/16:0/16:1), 48:2 (16:0/16:1/16:1) and 48:3 (16:1/16:1/16:1) were present throughout both E and S growth phases of *P. tricornutum* cells (Fig. 4A). An increase in the diversity of TAG molecular species was detected in Pt_OtElo5 cells, with 29 individual TAGs being present. Specifically, new highly unsaturated TAG species, 54:8, 54:9 and 56:8 were observed (Supplementary Fig. 4), with Pt_OtElo5 transgenic cells also showing significantly higher levels of 54:7 (Fig. 4A; Supplementary Fig. 5). DHA was incorporated in TAGs 52:7 (14:0/16:1/22:6), 54:7 (16:0/16:1/22:6), 54:8 (16:1/16:1/22:6), 54:9 (16:0/16:3/22:6) and 56:8 (16:1/18:1/22:6). It is interesting to note that DHA is predominantly present in TAG species containing “plastidial” fatty acids such as C16 acyl-chains. DHA-containing TAG molecular species 52:7, 54:8 and 54:9 were unchanged in abundance when cultures were shifted from exponential to stationary phase. Specifically the data demonstrates that in cells of Pt_OtElo5, levels of TAGs containing DHA averaged 12.5% in exponential stage and 10.5% in the stationary phase, which equates to an estimated threefold increase in comparison with Pt_WT (based on detector response; Fig. 4A). Thus, our data demonstrate the efficient channelling of transgene-generated DHA into TAG. This appears to be mediated by endogenous TAG biosynthetic activities but represents an obvious target for further enhancement, since DHA is known to accumulate in galactolipids of *P. tricornutum* (Yongmanitchai and Ward, 1993a, 1993b) as might other aspects of carbon assimilation and synthesis (Li et al., 2010).

### 3.5 Multigene expression in transgenic *P. tricornutum*

**Fig. 5:**
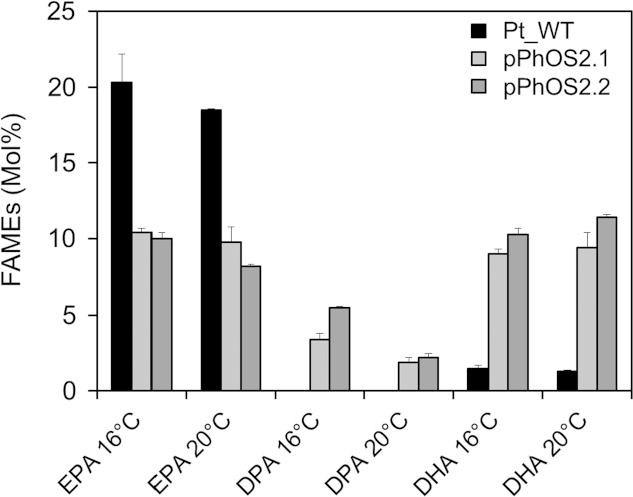
Co-expression of two heterologous omega-3 LC-PUFA biosynthetic activities in *P. tricornutum*. Fatty acid composition of Pt_WT, pPhOS2.1 (expressing OtElo5) and pPhOS2.2 (expressing OtD6Pt and OtElo5) cells during the S phase of growth at 16 °C and 20 °C. Values are the average of three experiments (± standard error).

To facilitate the expression of multiple heterologous genes in *P. tricornutum*, a new vector (designated pPhOS2-Supplementary Fig. 1) was constructed. This vector is based on previously described pPha-T1 vector (Zaslavskaia et al., 2000) and contains two multiple cloning sites (MCS) with unique restriction sites for inserting genes of interest. Each of these MCS is flanked by the promoter and terminator regions of the *FcpA* gene (Zaslavskaia et al., 2000) to promote the co-expression of two inserted genes. The coding sequence for *O. tauri* Δ5-elongase OtElo5 was inserted into position 1 of pPhOS2 vector and the resulting construct pPhOS2.1.1 was used to transform *P. tricornutum*. Cultures were grown at 20 °C and 16 °C under constant illumination (60 µmol photons m^−2^s^−1^). Multiple (5) independent zeocin-resistant colonies were obtained and used to inoculate cultures for further GC–MS analysis. The mean levels of DHA in analysed pPhOS2.1.1 strains was 9.0% (Supplementary Table 4), similar to levels previously observed with OtElo5 expression in pPHa-T1, confirming the functionality of this modified vector. The codon-optimised coding sequences for *O. tauri* Δ6-desaturase OtD6Pt was subsequently inserted into position 2 of construct pPhOS2.1.1, generating the two-gene (plus the selectable marker gene *ble*) pPhOS2.2.1 vector. This expression plasmid was introduced into *P. tricornutum* via biolistics and multiple independent zeocin-resistant colonies were obtained and used to inoculate cultures for further screening. Cultures were grown at 16 and 20 °C under constant illumination (60 µmol photons m^−2^ s^−1^). FAMEs analysis of transgenic strains expressing either single or double gene constructs revealed a further increase in DHA levels in transgenic strains co-expressing both OtElo5 and OtD6Pt, indicating the here-demonstrated potential for iterative metabolic engineering in *P. tricornutum* for high value lipid traits (Fig. 5; Supplementary Table 4).

## 4 Conclusions

We have successfully expressed Δ6-desaturase and Δ5-elongase activities from *O. tauri* in *P. tricornutum*, representing the first report of the metabolic engineering of the omega-3 trait in transgenic algae. Whilst transgenic strains expressing the Δ6-desaturase produced no significant alteration to the total omega-3 content, the expression of the Δ5-elongase dramatically increased the accumulation of DHA to eight-fold the levels present in WT strain. In addition, we carried out (for the first time in a transgenic diatom) co-expression of two heterologous enzyme activities, demonstrating further increases in DHA levels though simultaneous expression of OtElo5 and OtD6. The development of a simple vector for multigene expression opens the door to more complex metabolic engineering in *P. tricornutum*. Our data also indicate further opportunities to enhance the synthesis of DHA and its accumulation in TAG. For example, the presence of both EPA and DHA in the acyl-CoA pool represent potential substrates for acylation into neutral lipids, most obviously by the co-expression of the acyl-CoA:diacylglyceroltransferase (DGAT) activity which catalyses the addition of an acyl-CoA to the vacant sn-3 position of DAG to generate TAG. *P. tricornutum* contains several active DGAT genes (Gong et al., 2013; Guihéneuf et al., 2011), and experiments are underway to determine if this will further enhance the levels of DHA in TAG. Similarly, whilst we have shown that endogenous Δ4-desaturation is not a major bottleneck in the synthesis of DHA (via transgene-derived DPA), it is probable that transgenic co-expression of a Δ4-desaturase with the OtElo5 Δ5-elongase activity would result in further increased levels of DHA (and concomitant reduction in DPA). We believe that the combination of iterative metabolic engineering and lipidomics can help drive forward both our understanding of acyl metabolism in diatoms but also the establishment of *P. tricornutum* as an algal synthetic biology chassis to produce high value fatty acids such as omega-3 LC-PUFAs or medium chain fatty acids for biofuel (Radakovits et al., 2011).
