# Supplementary material for: Metabolic engineering of Phaeodactylum tricornutum for the enhanced accumulation of omega-3 long chain polyunsaturated fatty acids
Source: Metab Eng. 2014 Mar;22(100):3–9. doi: 10.1016/j.ymben.2013.12.003 (PMC3985434; doi:10.1016/j.ymben.2013.12.003)
Supplement: Supplementary file 1 — Supplementary data [file mmc1.pptx]

## Slide 1
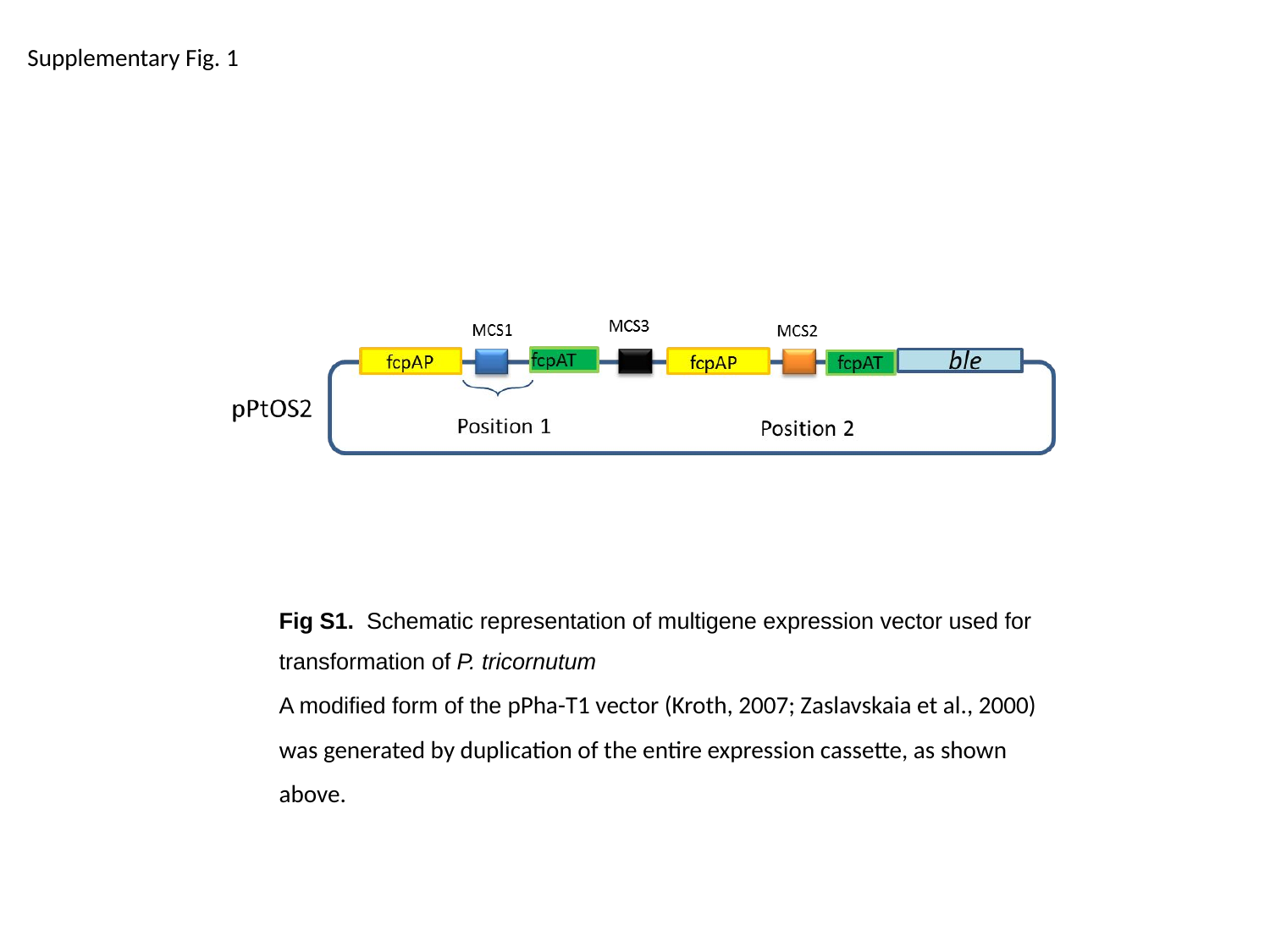

Supplementary Fig. 1
Fig S1. Schematic representation of multigene expression vector used for transformation of P. tricornutum
A modified form of the pPha-T1 vector (Kroth, 2007; Zaslavskaia et al., 2000) was generated by duplication of the entire expression cassette, as shown above.

## Slide 2
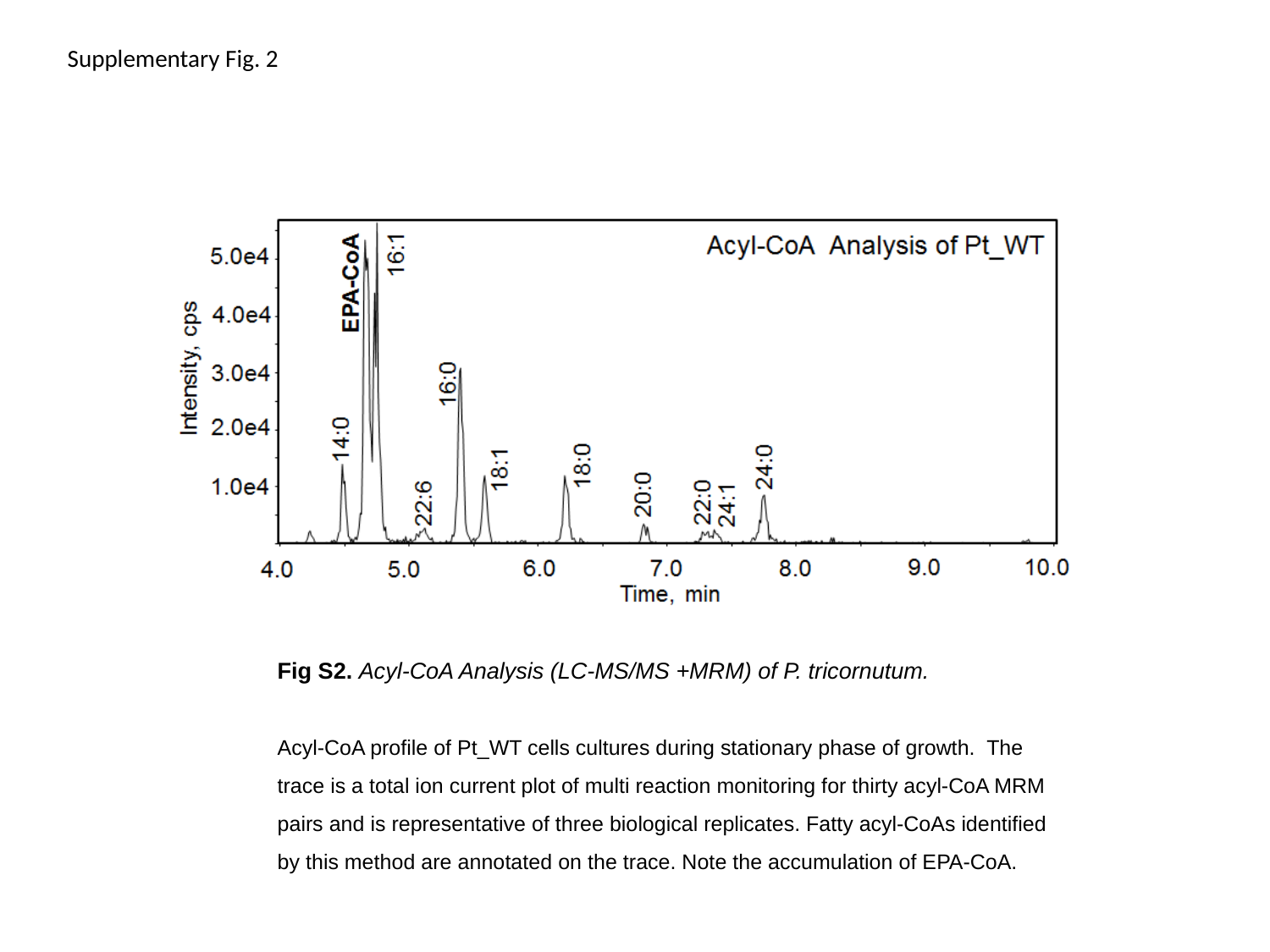

Supplementary Fig. 2
Fig S2. Acyl-CoA Analysis (LC-MS/MS +MRM) of P. tricornutum.
Acyl-CoA profile of Pt_WT cells cultures during stationary phase of growth. The trace is a total ion current plot of multi reaction monitoring for thirty acyl-CoA MRM pairs and is representative of three biological replicates. Fatty acyl-CoAs identified by this method are annotated on the trace. Note the accumulation of EPA-CoA.

## Slide 3
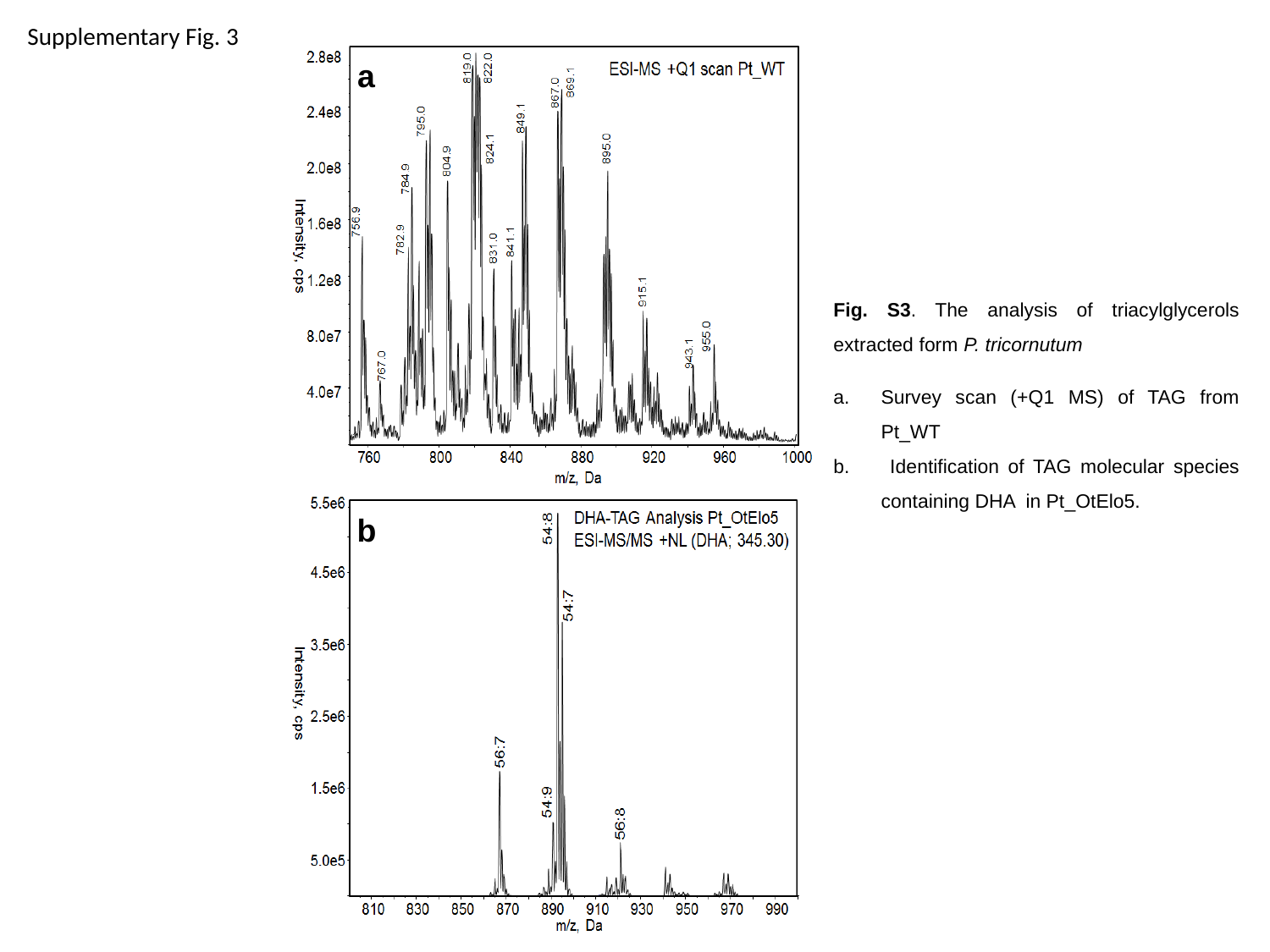

Supplementary Fig. 3
a
b
Fig. S3. The analysis of triacylglycerols extracted form P. tricornutum
Survey scan (+Q1 MS) of TAG from Pt_WT
 Identification of TAG molecular species containing DHA in Pt_OtElo5.

## Slide 4
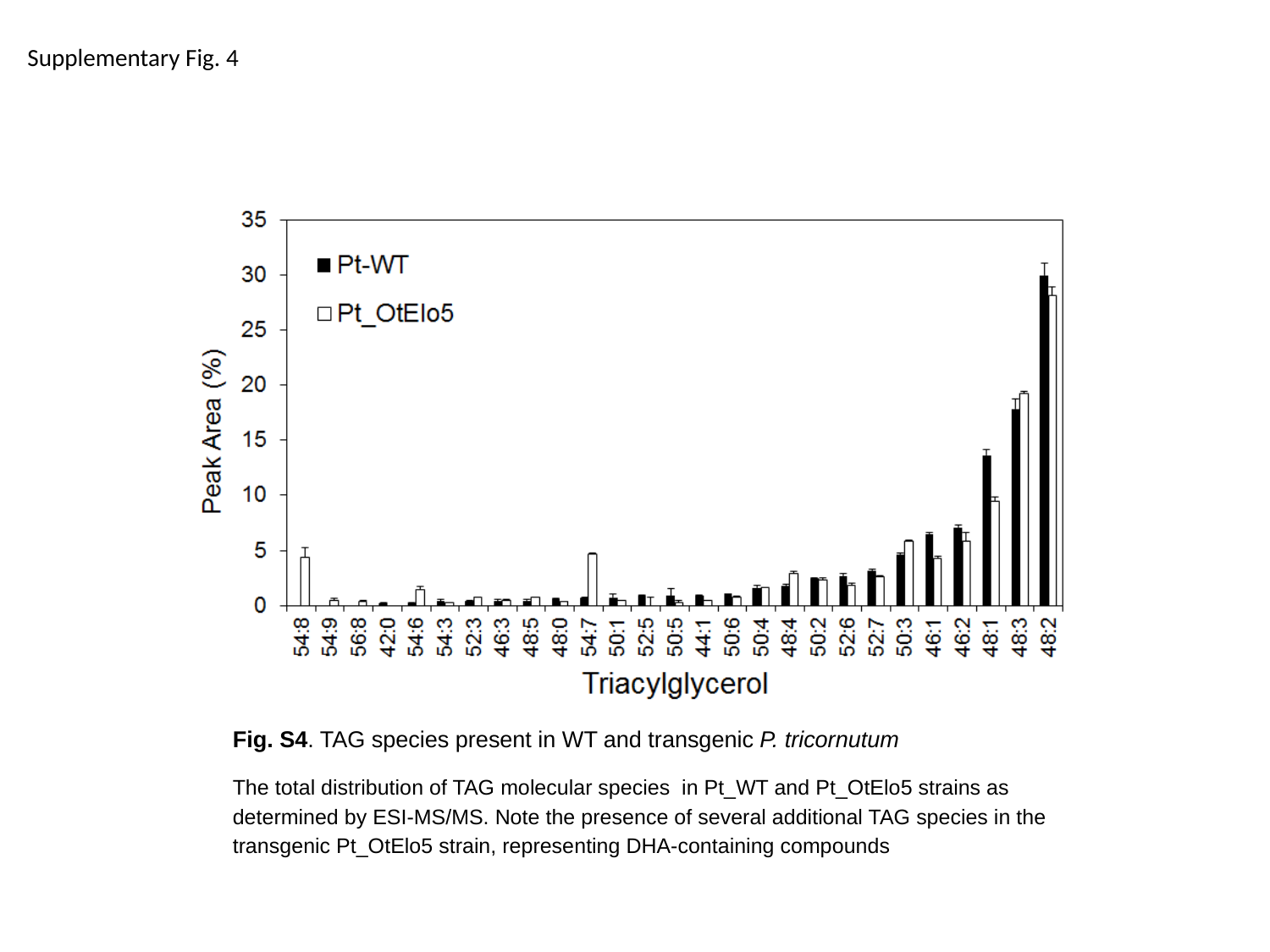

Supplementary Fig. 4
Fig. S4. TAG species present in WT and transgenic P. tricornutum
The total distribution of TAG molecular species in Pt_WT and Pt_OtElo5 strains as determined by ESI-MS/MS. Note the presence of several additional TAG species in the transgenic Pt_OtElo5 strain, representing DHA-containing compounds

## Slide 5
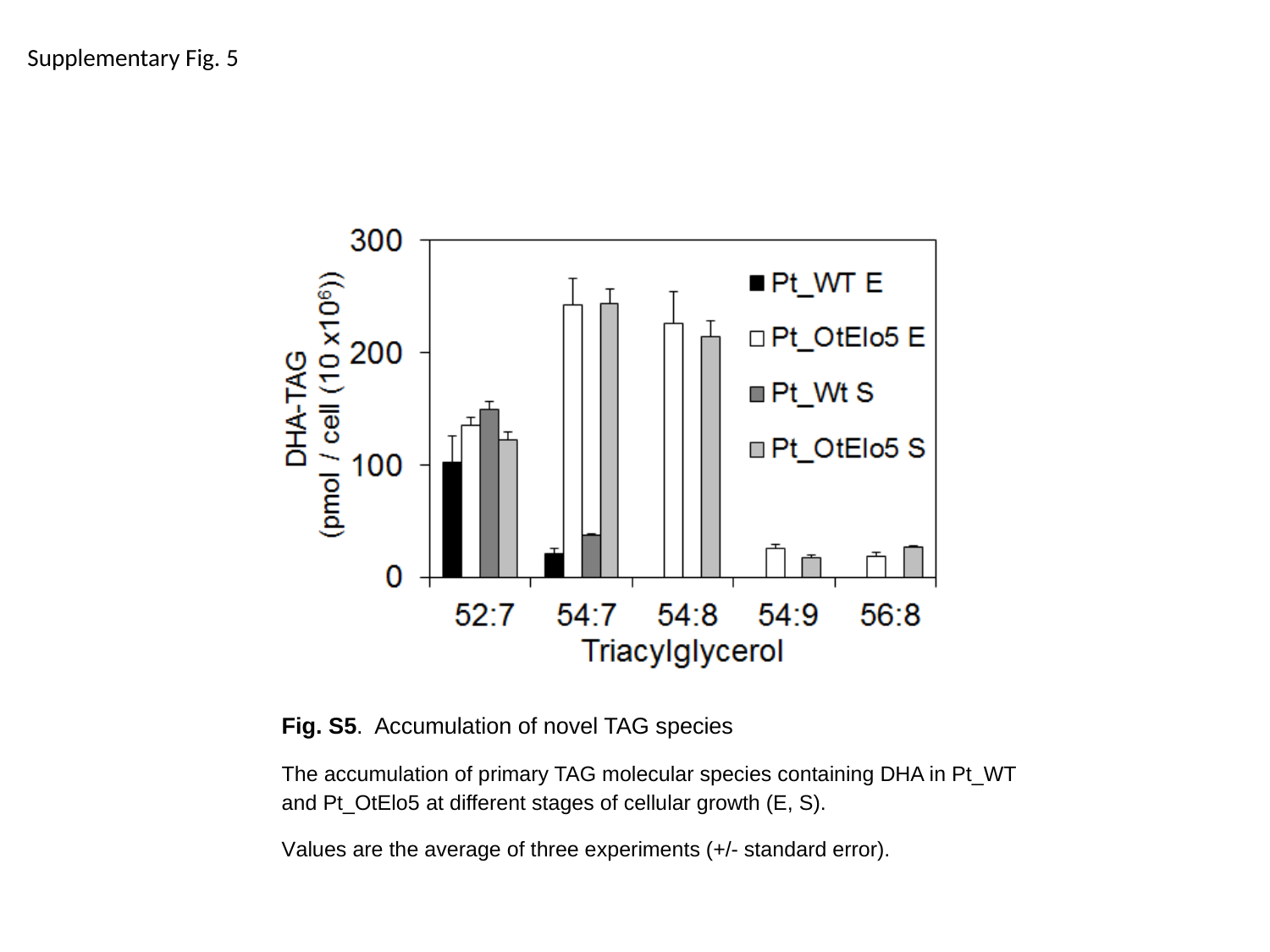

Supplementary Fig. 5
Fig. S5. Accumulation of novel TAG species
The accumulation of primary TAG molecular species containing DHA in Pt_WT and Pt_OtElo5 at different stages of cellular growth (E, S).
Values are the average of three experiments (+/- standard error).
