# Supplementary material for: Metabolic engineering of Phaeodactylum tricornutum for the enhanced accumulation of omega-3 long chain polyunsaturated fatty acids
Source: Metab Eng. 2014 Mar;22(100):3–9. doi: 10.1016/j.ymben.2013.12.003 (PMC3985434; doi:10.1016/j.ymben.2013.12.003)
Supplement: Supplementary file 2 — Supplementary data [file mmc2.docx]

**Supplementary Tables**

**Supplementary Table 1** Fatty acid composition (Mol %) of wild-type (Pt_WT) and transgenic *P. tricornutum* expressing Pt_OtD6N and Pt_OtD6Pt at 16^o^C and 20^o^C. Each measurement is the average of 3 biological replicates (± Standard Error). Nd = not detected.

| Fatty Acids  **14:0**  **16:0**  **16:1**  **16:3**  **18:0**  **18:1 n-9**  **18:1 n-11**  **18:4 n-7**  **20:5 n-3**  **22:5 n-3**  **22:6 n-3**  **24:0**  **Others** | Pt_WT  16°C 20°C | | Pt_OtD6N  16°C 20°C | | Pt_OtD6Pt  16°C 20°C | |
| --- | --- | --- | --- | --- | --- | --- |
|  | 5.3±0.2  22.3±1.0  39.2±1.6  0.8±0.4  0.5±0.1  6.8±0.1  2.2±0.1  1.0±0.1  20.3±1.9  *nd*  1.5±0.2  2.9±0.4  2.0±0.5 | 4.8±0.1  22.1±0.4  41.8±0.3  1.0±0.1  0.5±0.1  4.3±0.2  2.8±0.1  1.0±0.1  18.5±0.3  *nd*  1.3±0.1  2.4±0.1  1.9±0.1 | 6.2±0.1  20.8±0.1  36.6±0.3  1.0±0.1  0.9±0.1  6.7±0.7  3.0±0.1  0.8±0.2  22.0±0.2  *nd*  1.9±0.5  3.4±0.2  3.0±0.1 | 4.8±1.4  21.3±1.2  40.0±2.1  1.0±0.1  0.7±0.1  4.1±1.2  4.2±1.1  0.3±0.1  20.0±2.2  *nd*  1.8±0.1  3.4±0.1  4.8±0.2 | 6.0±0.3  20.5±0.1  37.2±1.0  0.8±0.4  0.6±0.1  6.6±0.2  2.6±0.1  1.4±0.1  22.9±1.0  *nd*  1.6±0.1  3.4±0.1  2.4±0.4 | 4.9±0.6  20.2±0.9  38.5±2.0  1.1±0.2  0.6±0.1  4.4±0.1  4.2±0.2  0.2±0.1  20.8±2.0  *nd*  1.5±0.2  3.0±0.5  4.4±0.1 |

**Supplementary Table 2.**

Fatty acid composition (Mol %) of wild-type and transgenic *P. tricornutum* expressing OtElo5 during exponential (E) and stationary (S) phases.

Cultures were grown at 20°C 60 µmol m-^2^s-^1^ under constant agitation at 70 rpm. Each measurement is the average of 3 biological replicates (± Standard Error).

| Fatty acids  **14:0**  **16:0**  **16:1**  **16:3**  **18:0**  **18:1**  **18:2 n-6**  **18:3n-6**  **18:4 n-3**  **20:5 n-3**  **22:5 n-3**  **22:6 n-3**  **24:0**  **Others** | Pt_WT  **E S** | | Pt_OtElo5  **E S** | |
| --- | --- | --- | --- | --- |
|  | 7.7±0.5  16.5±0.5  28.4±0.6  2.4±0.3  0.4±0.0  3.8±0.8  1.4±0.1  0.7±0.0  0.8±0.0  35.9±1.6  *nd*  2.0±0.3  5.2±0.2  1.8±0.3 | 4.8±0.5  22.1±0.6  41.8±0.5  1.0±0.0  0.5±0.0  7.3±0.2  0.6±0.0  0.6±0.0  1.0±0.0  18.5±0.4  *nd*  1.3±0.1  2.1±0.0  0.3±0.3 | 8.4±1.2  16.8±0.6  32.9±0.4  3.6±0.6  0.6±0.0  6.8±1.1  0.6±0.0  0.2±0.0  1.6±0.0  17.7±2.4  3.3±0.5  7.4±1.2  5.2±0.4  4.1±0.4 | 5.3±1.6  17.4±1.3  42.5±1.6  1.7±0.6  0.5±0.0  6.8±1.5  0.3±0.0  0.2±0.2  2.0±0.1  8.2±2.0  3.4±1.2  10.4±0.3  3.1±0.4  2.4±0.6 |

**Supplementary Table 3** Acyl-CoA composition of Pt_WT and Pt_OtElo5 strains.

Acyl-CoA composition (Mol %) of wild-type and transgenic *P. tricornutum* expressing OtElo5 during stationary phase. Cultures were grown at 20°C 60 µmol m-^2^s-^1^ under constant agitation at 70 rpm. Each measurement is the average of 3 biological replicates (± Standard Error).

| Acyl-CoA | Pt_WT | Pt_OtElo5 |
| --- | --- | --- |
| **14:0** | 4.5±0.40 | 5.8±0.53 |
| **16:0** | 21.9±2.23 | 15.8±0.85 |
| **16:1** | 19.7±1.84 | 29.0±0.95 |
| **16:3** | 0.2±0.03 | 0.4±0.03 |
| **18:0** | 4.5±0.63 | 2.4±0.22 |
| **18:1** | 6.5±0.68 | 4.3±0.43 |
| **18:2** | 0.9±0.09 | 0.9±0.06 |
| **18:3** | 0.7±0.12 | 0.3±0.03 |
| **18:4** | 0.7±0.08 | 1.0±0.12 |
| **20:0** | 0.9±0.19 | 0.8±0.09 |
| **20:1** | 0.2±0.03 | 0.3±0.05 |
| **20:4** | 3.5±0.20 | 2.6±0.14 |
| **20:5** | 31.2±1.35 | 23.10.66 |
| **22:0** | 1.3±0.13 | 1.6±0.17 |
| **22:4** | *nd* | 0.7±0.08 |
| **22:5** | 0.1±0.02 | 3.0±0.23 |
| **22:6** | 0.7±0.07 | 5.6±0.27 |
| **24:0** | 1.9±0.76 | 1.5±0.29 |
| **24:1** | 0.6±0.05 | 0.9±0.11 |

**Supplementary Table 4**. Fatty acid composition (Mol %) of wild-type (Pt_WT) and transgenic *P. tricornutum* expressing pPhOS2.1 and pPhOS2.2 at 16^o^C and 20^o^C. Each measurement is the average of 3 biological replicates (± Standard Error).

| Fatty Acids  14:0  16:0  16:1  16:3  18:0  18:1 n-9  18:1 n-11  18:4 n-7  20:5 n-3  22:5 n-3  22:6 n-3  24:0  Others | Pt_WT  16°C 20°C | | pPhOS2.1  16°C 20°C | | pPhOS2.2  16°C 20°C | |
| --- | --- | --- | --- | --- | --- | --- |
|  | 5.3±0.2  22.3±1.0  39.2±1.6  0.8±0.4  0.5±0.0  6.8±0.0  2.2±0.1  1.0±0.1  20.3±1.9  *nd*  1.5±0.2  2.9±0.4  2.0±0.5 | 4.8±0.1  22.1±0.4  41.8±0.3  1.0±0.1  0.5±0.1  4.3±0.1  2.8±0.1  1.0±0.1  18.5±0.1  *nd*  1.3±0.1  2.4±0.1  1.9±0.1 | 5.1±0.2  19.2±0.4  39.0±0.6  1.2±0.1  0.6±0.1  2.6±0.1  2.1±0.2  1.7±0.1  10.4±0.3  3.4±0.4  9.0±0.3  3.2±0.1  1.1±0.1 | 5.3±0.3  18.9±1.4  40.1±1.7  1.8±0.4  0.3±0.1  2.2±0.4  4.2±0.3  1.1±0.1  9.8±1.0  1.9±0.3  9.4±1.0  2.3±0.2  2.9±0.5 | 6.7±0.2  17.7±0.5  43.6±1.0  nd  0.5±0.0  1.2±0.6  2.7±0.1  1.6±0.0  10.0±0.4  5.5±0.1  10.3±0.4  3.3±0.1  2.9±0.3 | 6.3±0.1  18.4±0.3  40.6±0.5  2.0±0.1  0.3±0.1  0.6±0.4  3.7±1.0  1.1±0.1  8.2±0.1  2.2±0.3  11.4±0.2  2.2±0.8  3.2±0.2 |

|  |  |
| --- | --- |
